# Outcome and toxicity of radical radiotherapy or concurrent Chemoradiotherapy for elderly cervical cancer women

**DOI:** 10.1186/s12885-017-3503-2

**Published:** 2017-08-01

**Authors:** Weiping Wang, Xiaorong Hou, Junfang Yan, Jie Shen, Xin Lian, Shuai Sun, Zhikai Liu, Qingyu Meng, Dunhuang Wang, Mei Zhao, Jie Qiu, Ke Hu, Fuquan Zhang

**Affiliations:** Department of radiation oncology, Peking Union Medical College Hospital. Chinese Academy of Medical Sciences & Peking Union Medical College, NO.1 Shuaifuyuan Wangfujing, Dongcheng District, Beijing, 100730 People’s Republic of China

**Keywords:** Elderly cervical cancer, Radical radiotherapy, Chemoradiotherapy

## Abstract

**Background:**

Concurrent chemoradiotherapy (CCRT) is the standard treatment for local advanced cervical cancer. However, for elderly patients, studies are limited and the outcomes are controversial. We retrospectively analyzed the efficacy and tolerance of radical radiotherapy (RT) or CCRT in elderly cervical cancer patients and performed comparisons between them.

**Methods:**

We retrospectively analyzed the elderly cervical cancer patients (≥70 years old) treated with radical RT or CCRT between January 2006 and December 2014. For external beam radiotherapy, 50Gy in 25 fractions or 50.4Gy in 28 fractions were delivered via 3-dimensional conformal radiation therapy or intensity modulated radiation therapy. High-dose-rate intracavitary brachytherapy was performed with a dose of 30-36Gy in 5–7 fractions to point A. Concurrent chemotherapy regimens included weekly cisplatin and paclitaxel.

**Results:**

Seventy-three patients were eligible for this study. Twenty-one(28.8%) and 52(71.2%) patients suffered with FIGO stage IB-IIA and IIB-IVA disease, respectively. Twenty-four (32.9%) patients received CCRT. The median duration of follow-up was 32.4 months (4.8–118.8 months). The 3-year overall survival (OS), cancer-specific survival (CSS) and disease-free survival (DFS) were 64.9%, 67.8% and 66.5%, respectively. By multivariate analysis, CCRT was a significant predictive factor of OS(*p* = 0.023, 95% confidence interval [CI]: 1.172–8.860), CSS(*p* = 0.031, 95% CI: 1.131–13.908)and DFS(*p* = 0.045, 95% CI: 1.023 ~ 6.430). The 3-year OS of patients received RT and CCRT were 54.3% and 83.1%, CSS were 56.8% and 87.1%, DFS were 57.6% and 83.3%. There was no treatment related death. Grade 3–4 acute hematological, gastrointestinal and urinary toxicity incidences were 31.5%, 19.1% and 12.3%, respectively. For grade 3–4 chronic gastrointestinal and genitourinary toxicities, the incidences were 4.1% and 2.7%, respectively. Compared with RT, CCRT was related with high grade 3–4 hematological toxicity (16.3% and 62.5% respectively, *p* < 0.001), respectively. However, acute nonhematological toxicity and chronic toxicity were not significantly different.

**Conclusion:**

Elderly cervical cancer patients could tolerate radical RT and CCRT very well and get a favored survival. Compared with RT, CCRT could improve the survival of elder cervical cancer patients with similar nonhematological toxicity. CCRT should be considered in elderly cervical cancer patients.

## Background

In China, cervical cancer was the seventh most common cancer in women, with an estimated 98.9 thousands new cases and 30.5 thousands deaths in 2015 [1]. It is usually considered that cervical cancer is uncommon in elderly women. The incidence is similar from the third decade to 85 years old. However, the data from China showed that women older than 60 years old accounted for 23.8% of all the cervical cancer patients [1], probably associated with the aged tendency of population. Despite the dramatical decrease of incidence of cervical cancer in US during the recent years, incidence in elderly women has no significant reduction [2]. A more tough situation concerning is that the incidence of cervical cancer was still increasing in China [1].

Compared with young patients, elder patients are more likely to have more advanced disease, and receive less aggressive treatment. A research based on Surveillance, Epidemiology, and End Results (SEER) database showed that, for patients at the age of <50, 50–59, 60–69, 70–79 and ≥80 years old, the proportions of the International Federation of Gynecology and Obstetrics (FIGO) stage I patients were 70.1%, 49.2%, 45.7%, 39.9% and 33.2%, respectively, while proportions of patients with FIGO stage IIIB disease were 6.7%, 13.8%, 13.3%, 14.9% and 16.9%, respectively. Elderly patients were more frequently diagnosed with advanced diseased. For Stage IIB-IVA women, no treatment was allocated to 3.9% of patients <50 years old, compared with 7.3% of women aged 70–79 and 12.1% of the women ≥80 years old (*p* < 0.0001). The use of brachytherapy also declined with age (*p* < 0.0001, [3]. A research from Brazil also demonstrated that elderly patients were less likely to receive surgery, chemotherapy, brachytherapy, and more likely to receive no treatment [4].

For local advanced cervical cancer (stage IB2, IIA2, IIB-IVA), cisplatin-based concurrent chemoradiotherapy(CCRT) is the standard treatment at present [5]. However, elderly patients always suffer from weakness, malnutrition, comorbidity and so on, which may increase the incidence of treatment complications. Some elderly patients are unable to tolerate surgery or refuse surgery because of concerns about complications. Most elderly patients have no desire of reproduction or ovaries conservation. So some early stage patients may choose radical radiotherapy (RT) or CCRT as primary treatment other than surgery. Therefore, elderly patients have more demands for radical RT or CCRT. On the outcome and toxicity of CCRT for elderly patients, studies are limited at present [6–8].

In the present study, we retrospectively analyzed the efficacy and tolerance of elderly cervical cancer patients treated with radical RT or CCRT. We also performed comparisons between RT and CCRT.

## Methods

Cervical cancer patients treated with radical RT or CCRT in Peking Union Medical College Hospital (PUMCH) between January 2006 and December 2014 were retrospectively reviewed. The inclusion criteria were as follows: cervical cancer diagnosed by histopathology of biopsy; no evidence of distant metastasis; no previous surgery or radiotherapy for cervical cancer; ≥70 years old. Patients who underwent only palliative RT were excluded from this study.

### Radiotherapy

Patients underwent CT (16-slice Philips Brilliance Big Bore CT) simulation with intravenous and oral contrast agents; Rectum, bladder preparation and virginal marker were performed before CT simulation. The clinical target volume (CTV) and gross tumor volume (GTVnd) were contoured on the individual axial CT slices for each patient. The GTVnd included pelvic/para-aortic metastatic lymph nodes. The criteria for metastatic lymph nodes were: short diameter longer than 1 cm; proved by functional imaging technique like positron emission tomography or diffusion-weighted magnetic resonance imaging (MRI). The CTV covered the gross tumor, cervix, uterus, parametrium, upper part of the vagina to 3 cm below the tumor invasion, and regional lymph nodes (common iliac, internal iliac, external iliac, obturator, presacral, and/or para-aortic lymph nodes). A 5-mm margin was added to the GTV to create the final planning gross tumor volume (PGTVnd). The planning target volume (PTV) was defined as the CTV plus an 8-10 mm margin and an additional 5-10 mm margin to the cervix and uterus.

Three-dimensional conformal radiation therapy (3D–CRT) was delivered using 15- or 18-MV photons with a four-field box technique. A total dose of 50Gy (2Gy per fraction) was prescribed to the PTV. A 4-cm central shield was used after 36-40Gy to shield the rectum and bladder. Intensity modulated radiation therapy (IMRT) was delivered using 6-MV photons. A dose of 50.4Gy in 28 fractions was prescribed to the PTV. A concomitant boost to 59-61Gy was delivered to the PGTVnd for patients receiving IMRT. At least 95% of the final PTV or PGTV received 100% of the prescribed dose and at least 100% of the CTV or GTV was to receive 100% of the dose. For patients with IMRT, a second CT simulation and treatment planning were performed after 20 fractions external beam radiation therapy (EBRT).

The source used in intracavitary brachytherapy (ICBT) was iridium-192. ICBT generally began after 3 weeks of EBRT, 1–2 fractions per week. A cumulative dose of 30-36Gy/5–7 fractions was prescribed to point A according to International Commission of Radiation Units (ICRU) 38. Patients received conventional orthogonal film and brachytherapy planning after every insertion. CT was performed to check the applicator position after the first insertion.

### Chemotherapy

For patients receiving concurrent chemotherapy, the first line regimen was cisplatin (30–40 mg/m^2^/week). For patients with renal dysfunction, paclitaxel (60-80 mg/m^2^/week) was administered.

### Follow-up and evaluation of toxicity

Patients underwent gynecological examination and pelvic MRI/CT one month after treatment. After that, patients had follow-up examinations approximately every 3 months for the next 2 years, every 6 months for 3–5 years after treatment, and then once a year. The acute and chronic toxicity were evaluated with Common Terminology Criteria for Adverse Events (CTCAE) version 3.0.

### Statistical analysis

Survival rate was measured from the completion of treatment. Overall survival (OS), cause-specific survival (CSS) and disease-free survival (DFS) were estimated using the Kaplan–Meier method. The significance of difference was examined with a log-rank test. Cox’s proportional hazard model was used for the multivariate analysis. We used chi-square test, continuity correction and the Fisher exact test to compare the toxicity of radical RT and CCRT. Differences were considered statistically significant at *p* < 0.05. Statistical analysis was performed using SPSS v.19.0.

## Results

### Patient characteristics and treatment

During the time period from January 2006 and December 2014, there were 73 patients eligible for the research. The median age of patients was 74 years old, ranging from 70 to 88 years old. The majority of patients (68 patients, 93.2%) had squamous cell carcinoma, 4 patients (5.5%) with adenocarcinoma and 1 patient (1.4%) with small cell carcinoma. The FIGO stage ranged from IB to IVB. Nine (12.3%), 12 (16.4%) and 52 (71.2%) patients suffered with FIGO stage IB, IIA and IIB-IVA disease, respectively. There were 11 patients (15.1%) with positive lymph nodes, 4 (5.5%) of them with positive para-aortic lymph nodes. The detailed patients and tumor characteristics are shown in Table 1.

**Table 1:** Patients, tumor and treatment characteristics

	Characteristic	No. of patients	Percentage (%)
Age (years old)	Median	74	
	70–74 75–79 ≥80	44 20 9	60.3 27.4 12.3
Histology	Squamous cell carcinoma	68	93.2
	Adenocarcinoma	4	5.5
	Small cell carcinoma	1	1.4
Stage	IB	9	12.3
	IIA	12	16.4
	IIB	32	43.8
	IIIA	6	8.2
	IIIB	9	12.3
	IVA	5	6.8
LNM	Pelvic LNM	7	9.6
	Para-aortic LNM	2	2.7
	Pelvic and para-aortic LNM	2	2.7
External beam radiation therapy technique	3D–CRT	21	28.8
	IMRT	52	71.2
Dose of intracavitary brachytherapy	<30 Gy	19	26.0
	30-36 Gy	50	68.5
	>36 Gy	4	5.5
Completion of radiotherapy	Yes	70	95.9
	No	3	4.1
Duration of radiotherapy	≤8 weeks	58	79.5
	>8 weeks	15	20.5
Concurrent chemotherapy	No	49	67.1
	1-3 cycles	13	17.8
	≥4 cycles	11	15.1

Abbreviations: *LNM* Lymph nodes metastasis, *3D–CRT* 3-Dimensional conformal radiation therapy, *IMRT* Intensity modulated radiation therapy

3D–CRT and IMRT were performed to 21 (28.8%) and 52 patients (71.2%) respectively. The ICBT dose was 30Gy or higher in 54 patients (74.0%). The radiotherapy wasn’t completed in 3 patients. One of them didn’t complete radiotherapy because of grade 3 gastrointestinal(GI) toxicity, and this patient received extended-field RT and concurrent chemotherapy of cisplatin regimen. The other 2 patients treated with radical RT without concurrent chemotherapy refused to continue ICBT. One of them underwent radical surgery after 36Gy EBRT in 20 fractions and 6Gy ICBT in 1 fraction. The overall duration of radiotherapy ranged from 26 to 87 days with a median of 50 days. For patients received RT and CCRT, the median duration was 49 and 50 days respectively. The duration was more than 8 weeks for 15 patients (20.5%), including 11 patients (22.4%) treated with RT and 4 patients (16.7%) treated with CCRT.

Twenty-four patients (32.9%) received concurrent chemotherapy. Cisplatin was administered for 21 patients and paclitaxel for the other 3 patients. Eleven patients (15.1%) underwent ≥4 cycles weekly chemotherapy and the other 13 (17.8%) with 1–3 cycles. For these 13 patients, the reason for less than 4 cycles chemotherapy included acute hematological toxicity lasting several weeks (4 patients), grade 3 or higher GI toxicity (4 patients), renal failure (1 patients), refusing to continue chemotherapy without severe acute toxicity (4 patients). The treatment characteristics are detailed in Table 1.

### Outcome and pattern of failure

The median duration of follow-up was 32.4 months (4.8–118.8 months). The 3-year OS, CSS and DFS were 64.9%, 67.8% and 66.5%, respectively.

At the end of follow-up, 24 patients (32.8%) experienced tumor relapse. Eleven patients (15.1%) had locoregional failure, 9 patients (12.3%) experienced distant metastasis, and 4 (5.5%) had locoregional failure and distant metastasis. Of the 15 patients (20.5%) with locoregional failure, 6 patients didn’t achieve completely response (CR) after treatment. Nine patients experienced locoregional relapse after CR. For patients with locoregional failure, 2 patients had stage IIA disease, 5 patients with stage IIB disease, 1 patient with stage IIIA disease, 5 with stage IIIB disease and 2 patients with stage IVA disease. Among the 13 patients (17.8%) with distant metastasis, distant metastasis sites included lung (5 patients), para-aortic lymph nodes (3 patients), mediastinum lymph nodes (3 patients), liver (1 patient) and bone (1 patient).

Five (20.8%) of the 24 patients with tumor relapses (4 patients treated with CCRT and 20 patients treated with RT as initial treatment) received systemic chemotherapy for their relapses. One of the 4 patients (25%) treated with CCRT before received chemotherapy. As for the 20 patients treated with RT before, 4 patients (20%) underwent chemotherapy. The regimens included ciplatin, cisplatin and paclitaxel, cisplatin and fluorouracil.

At the time of last follow-up, 27 patients (36.9%) died. Twenty patients (27.4%) of them died of cervical cancer and 7 patients (9.6%) died of other causes, including heart failure (2 patients), meatus urinarius melanoma (1 patient), pulmonary embolism (1 patient), chronic obstructive pulmonary diseases (1 patient), cerebral hemorrhage (1 patient) and accidental death (1 patient).

### Prognostic factors

**Fig. 1:**
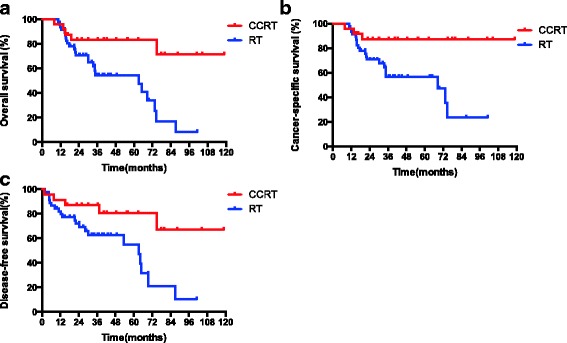
The overall survival (OS), cancer-specific survival (CSS) and disease-free survival (DFS) rates of elderly cervical cancer patients treated with radiotherapy (RT) and concurrent chemoradiotherapy (CCRT)

The results of univariate analysis are summarized in Table 2. The results indicated that FIGO stage, regional lymph nodes metastasis and CCRT were significant factors of OS, CSS and DFS. The 3-year OS of radical RT and CCRT were 54.3% and 83.1% (*p* = 0.0038), 3-year CSS were 56.8% and 87.1% (*p* = 0.0061), 3-year DFS were 57.6% and 83.3% (*p* = 0.0091), respectively. The OS, CSS and DFS curves of patients treated with RT and CCRT are shown in Fig. 1. The results of multivariate analysis are presented in Table 3. FIGO stage was an independent factor of DFS (*p* = 0.005, 95%confidence interval[CI]: 1.232–3.188). Regional lymph nodes metastasis were associated with worse OS (*p* = 0.001, 95% CI: 1.786–11.556), CSS (*p* < 0.001, 95% CI: 2.156 ~ 15.443) and DFS (*p* = 0.001, 95% CI: 1.817–11.017), CCRT was a significant predictive factor of OS (*p* = 0.023, 95% CI: 1.172–8.860), CSS (*p* = 0.031, 95% CI: 1.131–13.908) and DFS (*p* = 0.045, 95% CI: 1.023 ~ 6.430).

**Table 2:** Unvariate analysis of factors influencing OS, CSS and DFS

Factors	No.	3-year OS (%)	*p*	3-year CSS (%)	*p*	3-year DFS (%)	*p*
Age (years old)							
70–74	44	64.0	0.7516	65.8	0.8542	66.1	0.9648
≥75	29	65.8		70.0		67.5	
Histology							
Squamous cell carcinoma	68	65.9	0.3010	68.9	0.2478	67.3	0.3806
Other histology	5	50.0		50.0		60.0	
Stage							
I	9	88.9	0.0004	88.9	0.0074	88.9	<0.0001
II	44	72.6		72.6		74.8	
III	15	55.1		60.1		53.3	
IV A	5	20.0		0		0	
Lymph nodes metastasis							
Yes	11	27.3	<0.0001	0	<0.0001	18.2	<0.0001
No	62	75.1		78.7		75.5	
External beam irradiation technique							
3D–CRT	21	57.1	0.4969	61.2	0.5433	57.1	0.2360
IMRT	52	70.5		72.2		71.6	
Concurrent chemotherapy							
Yes	24	83.1	0.0038	87.1	0.0061	83.3	0.0091
No	49	54.3		56.8		57.6	

Abbreviations: *OS* Overall survival, *CSS* Cause-specific survival, *DFS* Disease-free survival, *LNM* Lymph nodes metastasis, *3D–CRT* 3-Dimensional conformal radiation therapy, *IMRT* Intensity modulated radiation therapy

**Table 3:** Multivariate analysis of factors influencing OS, CSS and DFS

Factors	OS		CSS		DFS	
	HR (95% CI)	*p*	HR (95% CI)	*p*	HR (95% CI)	*p*
Stage (I ~ IV)	1.539 (0.945 ~ 2.508)	0.083	1.370 (0.795 ~ 2.362)	0.257	1.982 (1.232 ~ 3.188)	0.005
Lymph nodes metastasis (Yes VS No)	4.543 (1.786 ~ 11.556)	0.001	5.770 (2.156 ~ 15.443)	<0.001	4.474 (1.817 ~ 11.017)	0.001
Concurrent chemotherapy(Yes VS No)	3.223 (1.172 ~ 8.860)	0.023	3.967 (1.131 ~ 13.908)	0.031	2.565 (1.023 ~ 6.430)	0.045

Abbreviations: *OS* Overall survival, *CSS* Cause-specific survival, *DFS* Disease-free survival, *HR* Hazard ratio, *CI* Confidence interval

### Toxicities

As shown in Tables 4, 25 patients (31.5%) experienced Grade 3 or higher acute hematological toxicity, the incidences in patients treated with RT and CCRT were 16.3% and 62.5%, respectively (*p* < 0.001). For 24 patients treated with CCRT, incidences of grade 3 or greater hematological toxicity of patients treated with 3D–CRT (7 patients) and IMRT (17 patients) were 71.4% and 58.8% (*p* = 0.908). There were 14 patients (19.1%) developed acute grade 3 or greater GI toxicity. The incidences were similar between RT (18.4%) and CCRT (20.8%). Nine patients (12.3%) experienced grade 3 or greater acute urinary toxicity. The incidence of patients treated with CCRT (16.7%) was higher than RT (10.2%). However, the difference wasn’t significant (*p* = 0.705).

**Table 4:** Acute and chronic toxicity of elderly cercal cancer patients treated with RT or CCRT

		Total	RT	CCRT	*p*
≥grade 3 acute toxicity	Hematological toxicity	25 (31.5%)	8 (16.3%)	15 (62.5%)	<0.001
	Gastrointestinal toxicity	14 (19.1%)	9 (18.4%)	5 (20.8%)	1.000
	Urinary toxicity	9 (12.3%)	5 (10.2%)	4 (16.7%)	0.705
≥grade 3 chronic toxicity	Gastrointestinal toxicity	3 (4.1%)	3 (4.1%)	0 (0%)	0.546
	Genitourinary toxicity	2 (2.7%)	1(2.0%)	1 (4.2%)	1.000

Abbreviations: *RT* Radiotherapy, *CCRT* Concurrent chemoradiotherapy

Four patients (5.5%) developed grade 3–4 chronic toxicity, including 1 patient with vesicorectal fistula, 1 patient with vaginorectal fistula, and 2 patients with proctitis. The patient with vesicorectal fistula suffered with stage IIB cervical cancer and was treated with 50.4Gy IMRT to the pelvic combine with 36Gy in 6 fractions ICBT. This patient developed vesicorectal fistula 15 months after treatment without cancer recurrence and received cystostomy and partial rectectomy. Another patient with stage IIIA disease was treated 50Gy in 25 fractions pelvic irradiation and 32.5Gy in 6 fractions ICBT combined with concurrent chemotherapy. The patient acquired CR after treatment and suffered with vaginal relapse 37.6 months after treatment. Then 30Gy in 15 fractions IMRT and 15Gy in 3 fractions ICBT were prescribed to the vaginal. The patient developed vaginorectal fistula 7 months after the second irradiation. She received surgery repair and had another 61.3 months disease-free survival.

The incidences of grade 3 or greater GI and genitourinary (GU) toxicity were 4.1% and 2.7%, respectively. The chronic GI and GU toxicity of CCRT was not significantly higher than RT.

## Discussion

It is controversial whether the survival of elderly cervical cancer patients is poorer than young patients. In retrospectively study with large population, the survival of elderly patients was always poorer than younger patients [3, 4, 9]. Maybe it was because elderly patients suffered from more advanced disease and treated with less aggressive treatment [3, 4, 9]. When treated similarly (such as RT or CCRT), the survival of young and elderly patients were similar [6, 10–12]. In this study, we compared the outcome of patients aged 70–74 and ≥75, the OS, CSS and DFS were not significantly different. This may indicate that, elderly patients can get a good survival if they received adequate treatment.

RT has been considered as an important primary treatment approach of cervical cancer for more than half a century. We have many experiences on the efficacy and tolerance of RT for elderly patients. It was reported that elderly patients could get equivalent survival to young patients when treated with RT [10–12]. A propensity score-matched study showed no significant differences in CSS, local failure and distant failure rates between elderly group (≥75 years) and young group (<60 years), although OS was worse in the elderly patients. The 5-year CSS of elderly and young groups were 73.1% and 76.2%, respectively (*p* = 0.456) [12]. Some studies reported that treatment toxicity was not significantly related with age [10, 11] while some others showed that age was a significant factor for radiation complications [12]. Ikushima, H, et al. repoted that the incidences of grade 2 or greater chronic toxicity was 22%, 31% and 8% respectively for patients aged ≤64、65–74 and ≥ 75 years old [10]. In the study of Wang YM et al., elderly patients experienced more grade 3 procitis compared with young patients (18.1% and 6.2%, respectively; *p* = 0.040 [12]. In our study, the incidences of grade 3 or greater acute hematological, GI and urinary toxicities were 16.3%, 18.4% and 10.2% respectively for patients treated with RT, which were comparatively low. Among 49 patients treated with RT, only 2 patients didn’t complete the treatment because of refusal to ICBT [10–12].

From 1999, several large randomized clinical trials proved that, compared with RT, the addition of cisplatin-based concurrent chemotherapy could improve the survival of cervical cancer [13]. These studies changed the treatment approach of cervical cancer. However, only very few of elderly cervical cancer patients were included in these clinical trials [13]. Elderly patients always can’t tolerate CCRT as good as young patients because of weakness, malnutrition, comorbidity and so on. However, because of the lacking clinical trials and guidelines, elderly patients were generally treated with the approach for young patients. The studies of CCRT were limited and controversial at present. Goodheart, M et al. compared 69 nonelderly cervical cancer patients (<65 years old) and 27 elderly patients (≥65 years old) treated with RT or CCRT. It demonstrated that the toxicity were similar between the two groups and CCRT was associated with decreased mortality (*P* < 0.01). The decrease in mortality did not differ between the two age cohorts (all causes: *p* = 0.66; cancer specific: *p* = 0.65) [6]. Chakraborty S, et al. treated 43 young cervical cancer patients (<65 years) and 23 elderly patients (≥65 years) with CCRT (98% of young patients and 65% of elderly patients) or RT. The EBRT was IMRT for all patients. Grade 3 hematological toxicities (26.7% versus 16.7%), GI toxicity (16.7% versus 13.3%), treatment breaks, treatment duration and early outcomes were not significantly different between young and elderly patients [7]. Park, J.H. et al. compared 61 elderly cervical cancer patients treated with RT and 44 elderly patients with CCRT. They found that CCRT was related with higher acute hematologic and gastrointestinal toxicity. However, the analysis showed no benefit of CCRT with respect to OS and CSS. The 5-year OS in RT group and CCRT group were 53.5% and 61.8% (*p* = 0.4534), CSS were 66.6% and 68.8% (*p* = 0.8584) [8].

In our study, CCRT was an independent predictive factor of OS (*p* = 0.023, 95% CI: 1.172–8.860), CSS (*p* = 0.031, 95% CI: 1.131–13.908) and DFS (*p* = 0.045, 95% CI: 1.023 ~ 6.430). For patients treated with RT and CCRT, the 3-year OS were 54.3% and 83.1%, 3-year CSS were 56.8% and 87.1%, 3-year DFS were 57.6% and 83.3%, respectively. This may indicate that CCRT could improve the survival of elderly cervical cancer patients. Since this was a retrospectively study with small population, we should interpret this result with caution.

Our study shown that CCRT was associated with more acute hematological toxicity. The acute nonhematological toxicity and chronic toxicity were similar between RT and CCRT. The incidences of grade 3 or higher hematological toxicity was 62.5% for patients treated with CCRT, which was higher than RT (16.3%, *p* < 0.001). The hematological toxicity was also much higher than previous studies in which most patients were non-elderly cervical cancer women. This may be becasuse the tolerance to CCRT of elderly patients is not as good as young patients. It was reported that, compared with 3D–CRT, bone marrow-sparing IMRT could reduce the dose of bone marrow and hematological toxicity [14–16]. In the study of Hui B et al., in IMRT group and 3D–CRT group, the V50 of bone marrow 9.79% and 15.4%, respectively. And the incidence of grade 2 or greater neutropenia was 80% and 40% in these 2 groups [14]. Considering the high hematological toxicity of CCRT, elderly patients may gain more benefits from bone marrow sparing IMRT. In our institute, we didn’t spare bone marrow for cervical cancer patients treated with IMRT. Incidence of grade 3 or greater hematological toxicity of 3D–CRT (71.4%) was higher than IMRT (58.8%) in our study. However, it was not significant.

For patients treated with CCRT, the incidence of grade 3 or higher acute GI and urinary toxicities were 20.8% and 16.7%, which were a little higher than RT (18.4% and 10.2%), although the difference was not significant. The grade 3 or greater chronic GI toxicity occurred in 4.1% and 0% (*p* = 0.546) of patients treated with RT and CCRT, respectively. For grade 3 or greater GU toxicity, the incidences was 2.0% and 4.2% (*p* = 1.000), respectively. CCRT didn’t significantly increase the chronic toxicity.

Generally speaking, 4–6 cycles concurrent chemotherapy were recommended for cervical cancer women treated with CCRT. In our stury, of 24 patients treated with CCRT, 13 patients received concurrent chemotherapy less than 4 cycles. In our institute this proportion was much higher than young patients. The main reason was treatment-related toxicity (9 patients, 69.2%). Three patients had not completed the radiotherapy, 2 of them were treated with RT and 1 with CCRT. The addition of concurrent chemotherapy didn’t influence the completion of radiotherapy. The acute toxicity of CCRT may lead to interruption of radiotherapy. And longer overall treatment time may lead to poor survival [17]. National Comprehensive Cancer Network (NCCN) guideline of cervical cancer recommended that radiotheray should be completed in 8 weeks [5]. In our study, concurrent chemotherapy didn’t prolong the duration of radiotherapy. The duration of radiotherapy was 49 and 50 days for patients received RT and CCRT. The duration of radiotherapy was longer than 8 weeks in 22.4% patients with RT and 16.7% patients with CCRT. Overall, most patients could tolerate CCRT very well.

During the last two decade, modern treatment approaches such as MRI based image guided brachytherapy (IGBT) and IMRT were used in the treatment of cervical cancer. Compared with 2D brachytherapy, IGBT could improve the survival with reduced severe morbidity [18–20]. A large multi-institutional study (RetroEMBRACE) involved 731 patients treated with ICBT combined with radio-chemotherapy. The study also included elderly patients. The median age was 53 years (range 23–91). IGBT combined with radio-chemotherapy leaded to excellent survival and limited severe toxicity. The 3-year LC, CSS and OS were 91%, 79% and 74%, respectively. The survival was much better than our study (3-year CSS and OS: 67.8% and 64.9%). This study also showed that concomitant chemotherapy had a significant impact on OS [18]. Compared with anteroposterior and posteroanterior parallel portals or 4 fields “box” radiotherapy, IMRT could decrease the treatment toxicity with comparable or better treatment efficiency [14–16, 21, 22]. Kidd et al. treated 452 cervical cancer patients with curative intent radiotherapy (135 with PET/CT guided definitive IMRT and 317 non-IMRT). IMRT group showed better OS and cause-specific survival (*p* < 0.0001). The incidences of ≥grade 3 bowel or bladder toxicity were 6% and 17% for patients treated with IMRT and non-IMRT, respectively (*p* = 0.0017) [21]. Considering the poor tolerance to CCRT, elderly cervical cancer patients may benefit more from new treatment approach like IGBT and IMRT.

Concurrent chemotherapy would significantly increase acute toxicity, which might be untolerated by some elderly cervical cancer patients. However, there is no guideline to recommend who should receive CCRT. Doctors may be confused about that. And this leads to some elderly patients who could tolerate CCRT receive less aggressively treatment. Comprehensive geriatric assessment (CGA) is a tool which covers assessment of ability to self-care, mobility and risk of falls, comorbidities, polypharmacy, nutritional status, cognitive function, psychological status, social support, and geriatric syndrome of elderly individuals. A CGA can predict morbidity and mortality in elder patients with cancer [23]. With a CGA, physicians can predict the benefit and risk of treatment and gave personalized treatment to individual patient. It was reported that, elderly cancer patients with prefrail/frail determined by a CGA-derived deficit-accumulation index were more likely to have ≥grade 3 chemotherapy toxicity [24]. Maybe because of the comparatively small population of elderly cervical cancer patients, the studies on the application of CGA in elderly cervical cancer patients are limited. For elderly local advanced cervical cancer patients, maybe CGA is an approach to look for the patients who could tolerate and benefit from CCRT.

## Conclusions

In our study, elderly cervical cancer patients could tolerate radical RT and CCRT very well and get a good survival. Compared with RT, CCRT could improve the survival of elderly cervical cancer patients with similar nonhematological toxicity. This indicated that CCRT should be considered for elderly cervical cancer patients. This study was a retrospective study. A prospective study is needed to determine the role of CCRT in this population. We also need more studies to look for the patients who could tolerate and benefit from CCRT in the future. CGA may be an approach to look for these patients.

## Abbreviations

3D–CRT: 3-Dimensional conformal radiation therapy
CCRT: Concurrent chemoradiotherapy
CGA: Comprehensive geriatric assessment
CI: Confidence interval
CR: Completely response
CSS: Cause-specific survival
CTCAE: Common Terminology Criteria for Adverse Events
CTV: Clinical target volume
DFS: Disease-free survival
EBRT: External beam radiation therapy
FIGO: International Federation of Gynecology and Obstetrics
GI: Gastrointestinal
GTVnd: Gross tumor volume
GU: Genitourinary
HR: Hazard ratio
ICBT: Intracavitary brachytherapy
ICRU: International Commission of Radiation Units
IMRT: Intensity modulated radiation therapy
MRI: Magnetic resonance imaging
NCCN: National Comprehensive Cancer Network
OS: Overall survival
PGTVnd: Planning gross tumor volume
PTV: Planning target volume
PUMCH: Peking Union Medical College Hospital
RT: Radiotherapy
SEER: Surveillance, Epidemiology, and End Results
